# DF-SSmVEP: Dual Frequency Aggregated Steady-State Motion Visual Evoked Potential Design with Bifold Canonical Correlation Analysis

**DOI:** 10.3390/s22072568

**Published:** 2022-03-27

**Authors:** Raika Karimi, Arash Mohammadi, Amir Asif, Habib Benali

**Affiliations:** 1 Department of Electrical and Computer Engineering, Concordia University, 1455 De Maisonneuve Blvd. W. EV-009.187, Montreal, QC H3G 1M8, Canada; r_arim@encs.concordia.ca (R.K.); habib.benali@concordia.ca (H.B.); 2 Concordia Institute for Information System Engineering, Concordia University, 1455 De Maisonneuve Blvd. W. EV-009.187, Montreal, QC H3G 1M8, Canada; 3 Department of Electrical Engineering and Computer Science, York University, Toronto, ON M3J 1P3, Canada; asif@eecs.yorku.ca

**Keywords:** steady-state motion evoked potentials, EEG signals, Brain Computer Interfaces

## Abstract

Recent advancements in Electroencephalographic (EEG) sensor technologies and signal processing algorithms have paved the way for further evolution of Brain Computer Interfaces (BCI) in several practical applications, ranging from rehabilitation systems to smart consumer technologies. When it comes to Signal Processing (SP) for BCI, there has been a surge of interest on Steady-State motion Visual Evoked Potentials (SSmVEP), where motion stimulation is used to address key issues associated with conventional light flashing/flickering. Such benefits, however, come with the price of being less accurate and having a lower Information Transfer Rate (ITR). From this perspective, this paper focuses on the design of a novel SSmVEP paradigm without using resources such as trial time, phase, and/or number of targets to enhance the ITR. The proposed design is based on the intuitively pleasing idea of integrating more than one motion within a single SSmVEP target stimuli, simultaneously. To elicit SSmVEP, we designed a novel and innovative dual frequency aggregated modulation paradigm, called the Dual Frequency Aggregated Steady-State motion Visual Evoked Potential (DF-SSmVEP), by concurrently integrating “Radial Zoom” and “Rotation” motions in a single target without increasing the trial length. Compared to conventional SSmVEPs, the proposed DF-SSmVEP framework consists of two motion modes integrated and shown simultaneously each modulated by a specific target frequency. The paper also develops a specific unsupervised classification model, referred to as the Bifold Canonical Correlation Analysis (BCCA), based on two motion frequencies per target. The corresponding covariance coefficients are used as extra features improving the classification accuracy. The proposed DF-SSmVEP is evaluated based on a real EEG dataset and the results corroborate its superiority. The proposed DF-SSmVEP outperforms its counterparts and achieved an average ITR of 30.7 ± 1.97 and an average accuracy of 92.5 ± 2.04, while the Radial Zoom and Rotation result in average ITRs of 18.35 ± 1 and 20.52 ± 2.5, and average accuracies of 68.12 ± 3.5 and 77.5 ± 3.5, respectively.

## 1. Introduction

Recent advancements in Electroencephalographic (EEG) sensor technologies and Signal Processing (SP) algorithms have paved the way for further evolution of Brain Computer Interface (BCI) systems [1]. The ultimate goal of a BCI system is to establish a robust communication channel with high throughput and accuracy between brain and the outer world. BCI systems have found several practical applications ranging from rehabilitation/assistive systems to diagnosis/prognoses of neurological disorders [2,3,4]. Recent technology trends show that leading technology companies are racing to develop advanced BCI systems coupled with Augmented Reality (AR) visors. It is widely expected that AR coupled with BCI would be the next era of computing. In this regard and for integration within an AR environment.

Literature Review: Generally speaking, there are two main visual BCI Paradigms, (1) Steady-State Visually Evoked Potential (SSVEP) [5,6,7,8,9,10,11,12,13,14], where light flashing (flickering) visual stimulus is used to induce evoked potentials in the EEG signals, and; (2) Steady-State motion Visual Evoked Potentials (SSmVEP) [15,16,17,18,19], where instead of using flickering, some form of graphical motion is used to evoke potentials. The former category (SSVEP) has been the main research theme due to its high achievable Information Transfer Rate (ITR), minimal requirement for user training, and excellent interactive potentials, such as high tolerance to artifacts and robust performance across users. However, flickering light causes extensive mental stress. Continuous use of SSVEPs (looking at flickering patterns for a long period of time), therefore, may cause seizure or eye fatigue.The second category (SSmVEP) is introduced to address these issues while keeping all the aforementioned benefits of the SSVEPs.

One issue that is shared by both categories is the challenge of coding more targets with available resources. Within the context of SSVEPs, the following research works were conducted to address this issue: Reference [12] introduced simultaneous phase and frequency modulations. Reference [13] used modulation in time, i.e., lengthening of the trial duration. Two different flickering-frequencies are shown consecutively for each target. Following a similar path, Reference [10] focused on using more than one frequency in a single target (but not at the same time) via Frequency-Shift Keying (FSK) modulation, also referred to as code modulation, i.e., trial time is again used for modulation purposes. Similarly, Reference [11] used code modulation with a single frequency for each target but with different phase shifts over one trial to enhance the system. Such code VEPs [10,11,14] and phase modulation [11,12] techniques are, however, very sensitive to synchronization, and as a trial time is used for modulation purposes, ITR will be compromised. When it comes to SSmVEPs, the issue of coding more targets with enhanced ITR has not yet been considered; this paper addresses this gap.

**Figure 1:**
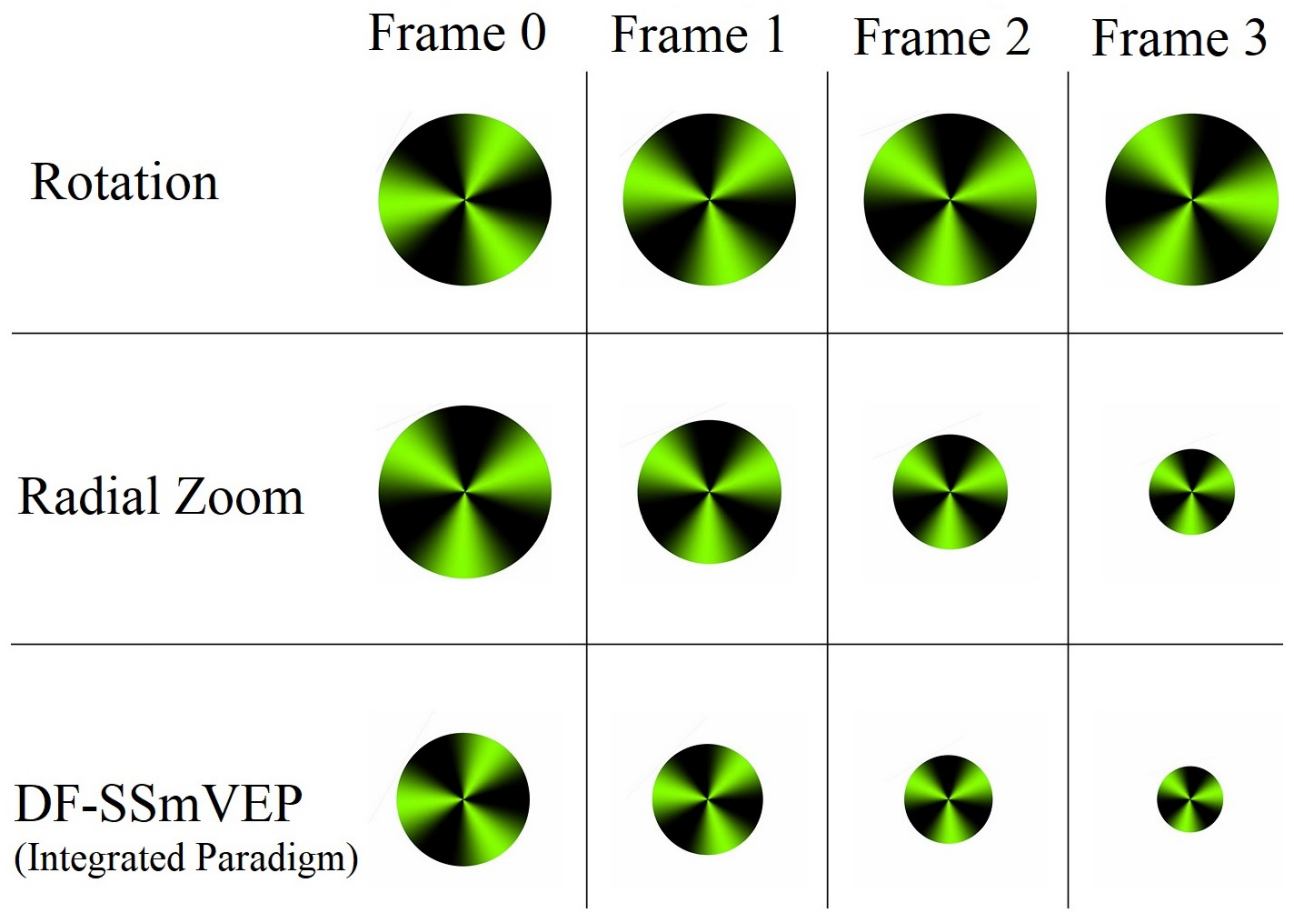
Proposed DF-SSmVEP paradigm developed by concurrent inclusion of two types of the motion (rotation and resizing).

**Figure 2:**
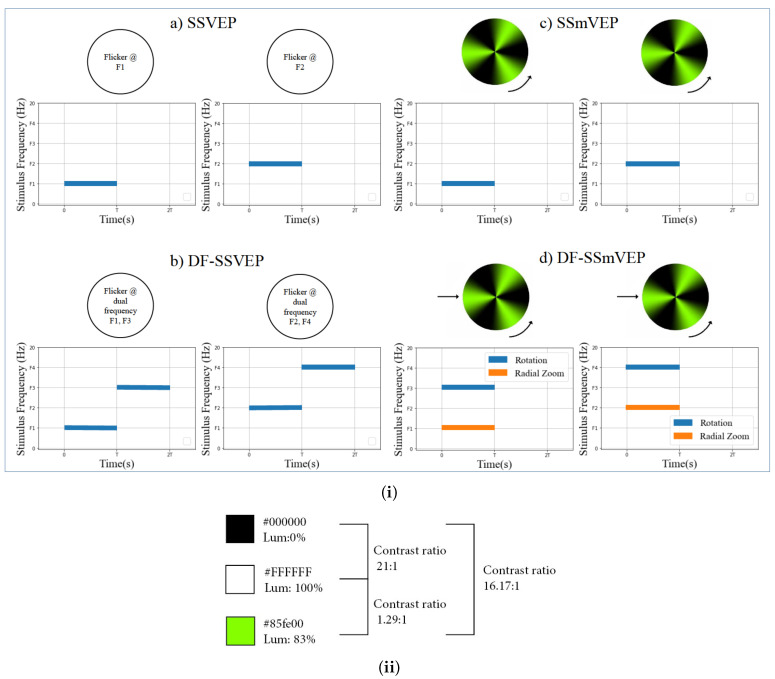
(**i**) Comparison between existing SSVEP frequency modulation schemes (**a**,**b**) with SSmVEP (**c**), and the proposed DF-SSmVEP (**d**). (**ii**) Luminance contrast ratio of the colors used in the designed DF-SSmVEP.

Contributions: To address the aforementioned problem, we focus on designing a novel SSmVEP paradigm without using additional resources such as trial time, phase, and/or number of targets to enhance the ITR. The proposed design is based on the intuitively pleasing idea of using more than one simultaneous motion within a single SSmVEP target stimuli. More specifically, as shown in Figure 1, to elicit SSmVEP we designed a novel and innovative dual frequency aggregated modulation paradigm, referred to as the DF-SSmVEP, by concurrently integrating “Radial Zoom” and “Rotation” motions in a single target without increasing the trial length. Figure 2i visually compares four different paradigms: Conventional SSVEP frequency modulation is shown in Figure 2(ia), where two target frequencies, “F1” and “F2”, are evoked in two different trials via flickering. Figure 2(ib) is similar to Figure 2(ia) where now two target frequencies are used together, one after another by increasing the trial time. Figure 2(ic) illustrates two SSmVEP modulations similar to Figure 2(ia), but target frequencies are evoked now via motion of the circle. Figure 2(id) shows the proposed DF-SSmVEP design where now two target frequencies are used together simultaneously eliminating the need to increase (sacrifice) the trial length for achieving higher accuracy as is the case in code/frequency modulated SSVEPs [10,11].

The paper also develops a specific unsupervised classification model adopted to the proposed innovative DF-SSmVEP paradigm. More specifically, in contrast to existing works, we propose an unsupervised SSmVEP detection technique, called the Bifold Canonical Correlation Analysis (BCCA) using unique characteristics of the proposed dual aggregated frequency design. The BCCA exploits the availability of two motion frequencies for each target and separately considers each single frequency of the targets as a reference. The corresponding covariance coefficients are then used as extra features advancing the classification accuracy. The proposed DF-SSmVEP is evaluated based on a real EEG dataset.

## 2. The Proposed DF-SSmVEP

In this section, we present the proposed DF-SSmVEP framework. The designed DF-SSmVEP stimulation paradigm includes a green and black circle with two motion modes. The first motion is the “Radial Zoom” in which the size of the circle changes periodically. The second mode is the “Reciprocal Rotation” of the circle between −45${}^{\circ }$ and 75${}^{\circ }$. Radial zoom motion and rotation motion are selected as candidates for integration following previous evaluations [19,20]. Generally speaking, the motion inversion frequency is defined as the frequency with which the direction of the current motion is changing within the reciprocal motion. Furthermore, the SSmVEP frequency is equal to the stimulation’s frequency, which is related to the aforementioned motion inversion frequency. When it comes to the design of the DF-SSmVEP paradigm, we had to decide on the motion choices. In this regard, we followed the intuition provided by Reference [20], i.e., to create a stimuli for evoking SSmVEPs, we can use any paradigm that has periodically changing motion. The two designs are integrated such that the focal point of one paradigm is overlaid with that of the second one. In the proposed DF-SSmVEP paradigm, the focal point will be the center of the black-green circle, i.e., the center of oscillation for the two segments of the design (resizing and oscillation of circle). To make the proposed design as efficient as possible and to reduce fatigue [21], the proposed DF-SSmVEP design does not include high contrast colors, improving the practical applicability of the SSmVEP stimuli. As shown in Figure 2(ib), the Luminance Contrast Ratio (LCR) associated with our green-black paradigm is lower than that of the conventional black-white design.

In the design stage and for selecting the underlying targeted frequencies, one needs to take into account the refresh rate of the monitor over which the SSmVEP stimuli is shown. Essentially, the monitor’s refresh rate is a restricting factor limiting the number of frames that can be shown within each motion direction change. According to Reference [21] and for achieving flexibility in the implementation of target frequencies, the underlying paradigms should have a different (variable) number of frames within each cycle. In the implemented setting, the refresh rate of the monitor was 60 Hz, based on which initially we designed the number of frames within each half of a cycle in an asymmetric fashion. We refer to this initial step as binary stimulus sequencing. More specifically, within each half of a cycle of the DF-SSmVEP, the number of frames is constructed as follows

(1) $$\begin{array}{c} S\left(f^{\left(T\right)},i\right)=\mathrm{square}\left[2\pi f^{\left(T\right)}\left(\frac{i}{R_{r}}\right)\right], \end{array}$$

where the target frequency is denoted by *f*; Refresh rate of the monitor is denoted by $R_{r}$; *i* is used as the frame index. Finally, the stimulus sequences corresponding to *f* are represented by S(f,i). It is worth noting that within each half of a cycle, an expansion or contraction is performed, which is equivalent to half of the reciprocal motion. Therefore, we can generate stimulus with oscillations up to $f\leq R_{r}/k$ around the SSmVEP’s target frequency. More specifically, we need a minimum number of *k* frames over half of a cycle to have an understandable and comfortable SmVEP paradigm.

Coding Algorithm: Assume that the maximum number of targets (objects shown on the screen simultaneously) is denoted by $N^{\left(T\right)}$. In other words, $N^{\left(T\right)}$ target frequencies are selected within the limited frequency spectrum of [$f_{\min},f_{\max}$] available for constructing the stimuli. $f_{i}$, for ($1\leq i\leq N^{\left(T\right)}$), represents the target frequency for the ith target/object assumed to be sorted in an ascending order, i.e., ($f_{\min}\leq f_{1}<f_{2}<\ldots <f_{N^{\left(T\right)}}\leq f_{\max}$). These $N^{\left(T\right)}$ target frequencies need to be derived in an intelligent fashion such that the best performance among all the susceptible frequencies is achieved (i.e., achieve accuracy improvements without reducing the ITR). In the proposed dual aggregated design, each target includes two motions with two distinct frequencies. These frequencies are assigned to targets in which no two pairs of targets have more than one adjacent frequencies. More specifically, the objective of the coding algorithm is to find these two underlying target frequencies in such a way that each pair of objects at most have one adjacent target frequency. For each consecutive frequencies $f_{i}$ and $f_{i+1}$, $g_{i}$ is defined as

(2) $$g_{i}=\left\{\begin{array}{c} \frac{f_{i}+f_{i+1}}{2},\;\;\;\;\;\forall i\in \left[1,N-1\right]\\ f_{i}+M,\;\;\;\;\;\;\;\;\;\;i=N \end{array}\right.$$

where $M=\min[\frac{f_{i+1}-f_{i}}{2}]$∀i∈[1,N−1]. Each $g_{i}$ is adjacent to $f_{i}$ and $f_{i+1}$. More specifically, consider $P=\{\left(a_{i},bi\right)\}$, for (1≤i≤N), representing the set of N≥5 target pairs where $a_{i}$ and $b_{i}$ are the new SSmVEP frequencies used for the *i*th object.s $a_{i}$ and $b_{i}$ in *P* are defined as follows:

(3) $$a_{i}=\left\{\begin{array}{c} f_{1},\;\;\;\;\;i=1\\ f_{i+1},\;\forall i\in \left[2,N-1\right]\\ f_{2},\;\;\;\;\;i=N \end{array}\right.\;b_{i}=\left\{\begin{array}{c} g_{N-1},\;\;\;\;\;i=1\\ g_{i-1},\;\;\;\;\;\;\forall i\in \left[2,N-1\right]\\ g_{N},\;\;\;\;\;\;\;\;\;\;i=N \end{array}\right..$$

Pre-processing: The proposed SSmVEP paradigm is implemented via a BCI system for real EEG data collection. In this regard, the first step is pre-processing of EEG signals associated with the proposed SSmVEP paradigm, as collected EEG signals are, typically, contaminated by high/low noises and/or artifacts. In this context, one needs to apply time-domain and/or spatial filters on the EEG signals to remove the artificats/noises and allow efficient extraction of SSmVEP related features. To filter high-frequency noises and artifacts, we applied a zero-phase Chebyshev Type I bandpass filter, which passes frequencies within the rage of 2–40 Hz.

### Proposed BCCA Paradigm

To learn potential linear relations that exist between two multi-dimensional EEG signals, typically, the Canonical Correlation Analysis (CCA) is used. The CCA is a statistical approach that aims at extracting two linear projection vectors in such a way that the resulting linear combination of the two original EEG signals has maximum correlation coefficient. More specifically, consider that the two original EEG signals are represented by matrices X and *Y*. Then, the objective is to compute projection vectors $w_{x}$ and $w_{y}$ that results in a maximized correlation coefficient, denoted by ρ between $w_{x}^{T}X$ and $w_{y}^{T}Y$. This process can be performed as follows:

(4) $$\rho =\max\frac{E(w_{x}^{T}XY^{T}w_{y})}{\sqrt{E\left(w_{x}^{T}XY^{T}w_{x}\right)E\left(w_{y}^{T}XY^{T}w_{y}\right)}}.$$

Reference signals are constructed at the stimulation frequency $f_{i}$ as

(5) $$y_{i}={\left[\cos2\pi f_{i}t,\sin2\pi f_{i}t,\ldots ,\cos2\pi N_{h}f_{i}t,\sin2\pi N_{h}f_{i}t\right]}^{T},$$

where $t=\frac{1}{f_{s}},\ldots ,\frac{m}{f_{s}}$ with *m* denoting the sample point; The sampling rate is represented by $f_{s}$, and; The number of harmonics is represented by $N_{h}$. It is worth noting that the value of $N_{h}$ depends on the designed paradigm and varies from one scenario to another. Therefore, we used the Welch Power Spectrum of the underlying signals and computed $N_{h}$ experimentally. Consider X as the matrix of the EEG signals collected from *K* different channels. The CCA finds linear combination of coefficients with the largest correlation between X and Y. We would like to mention that use of CCA for comparing SSVEP and SSmVEP paradigms, which evoke specific frequency range and harmonics within EEG signals, is common in the literature [19,22]. These targeted frequencies are dominant in the signal harmonics and other components can be approximately modeled by linear noise. In this model, the collected signals from the involved regions, Occipital lobe, contain both evoked SSVEP harmonics and other artifacts. Moreover, the goal of CCA is increasing the Signal to Noise Ratio (SNR) and removing background EEG activities to detect evoked Signals. The standard CCA method performs canonical correlation analysis between multi-channel EEG signals and predefined sinusoidal reference signals at stimulation frequencies and then identifies the target frequency based on the canonical correlation values.

In the BCCA fusion, there is a feature vector for each sample concerning each target frequency. Contemplating the Power Spectral Density (PSD) of the EEG signal, collected during the aggregated paradigm shows that only one of the peaks is significant for some trials, i.e., one of the two modulated frequencies has more impact on the visual pathways of the brain. To capitalize on this unique property and enhance the DF-SSmVEP, the following three references are incorporated to create the feature vector

(6) $$\begin{array}{ccc} y_{1} & = & {\left[\cos\left(2\pi f_{i,1}t\right),\sin\left(2\pi f_{i,1}t\right),\ldots ,\cos\left(2\pi N_{h}f_{i,1}t\right),\sin\left(2\pi N_{h}f_{i,1}t\right)\right]}^{T} \end{array}$$

(7) $$\begin{array}{ccc} y_{2} & = & {\left[\cos\left(2\pi f_{i,2}t\right),\sin\left(2\pi f_{i,2}t\right),\ldots ,\cos\left(2\pi N_{h}f_{i,2}t\right),\sin\left(2\pi N_{h}f_{i,2}t\right)\right]}^{T} \end{array}$$

(8) $$\begin{array}{ccc} y_{c} & = & C(y_{1},y_{2},{\left[\cos\left(2\pi \left(f_{i,1}+f_{i,2}\right)t\right),\sin\left(2\pi \left(f_{i,1}+f_{i,2}\right)t\right)\right]}^{T},2) \end{array}$$

where $f_{i,j}$ represents the *j*th stimulation frequency of *i*th target, and the operator C(a,b,c,2) concatenates three matrixes a, b and c vertically. The projection of each vector is separately calculated leading to three different weight vectors between test signal ***X*** and: (i) ($w_{y_{1}},w_{X_{1}}$) sine/cosine reference of first frequencies; (ii) ($w_{y_{2}},w_{X_{2}})$ reference of second frequency of targets, and; (iii) ($w_{y_{c}},w_{X_{c}}$) sine/cosine reference of both frequencies of targets. The feature vector v is

(9) $$\begin{array}{c} v={\left[\rho_{1},\rho_{2},\rho_{c}\right]}^{T},\;\mathrm{and}\;\rho_{a}=\frac{\rho_{1}+\rho_{2}+\rho_{c}}{3}, \end{array}$$

where $\rho_{a}$ is used as the final value to represent the correlation between the unknown sample and the frequencies of a target. It is worth mentioning that the proposed BCCA is an unsupervised technique as such there is no need for a separate training step. Consequently, all the available trials of sessions are used in the testing stage.

## 3. Experimental Results

In this section, we present different experimental evaluations based on a real EEG dataset collected for this study. We aim to evaluate the performance and effectiveness of the proposed DFSSmVEP paradigm and also compare it with its conventional SSmVEP counterparts, i.e., Radial Zoom and Rotation, when applied independently. We would like to mention that another possibility would be to consider comparisons with conventional SSVEP (flickering) paradigms. However, SSVEPs are inherently categorized as a different group of VEPs because of the induced level of mental load and fatigue. As such and following the common practice, we have not included results for conventional SSVEPs.

### 3.1. Experimental Setup

To evaluate the performance of the proposed DF-SSmVEP framework, we created a real EEG dataset from ten healthy individuals (five men and five women). Among the ten participants (each between 20 and 27 years old), none had any evidence of color or visual-recognition ailment issues, but five had prior experience with use of EEG signals and have participated previously in BCI experiments. We would like to mention that, for data collection, we followed the common, standard, and accepted approach in the BCI domain (e.g., [20]) where typically 9–11 subjects are used, and the trial length ranges from 2 s to 6 s. The data were collected with the policy certification number 3007997 of Ethical acceptability for research involving human subjects approved by Concordia University. We used g.Nautilus, which is a wireless and portable bio-signal acquisition headset from g.tech Medical Engineering to collect the required EEG signals from the ten participants. The used g.Nautilus headset has sampling rate of 500 Hz and consists of 32 active, bipolar, and wet electrodes. Following the conventional approach, we placed the reference electrode at the earlobe, and the ground electrode at the frontal position (Fpz). To collect the EEG signals, we focused on the parietal-occipital area of the brain and used signals from the following electrodes: $P_{o3}$, $P_{o4}$, $P_{o7}$, $P_{o8}$, $P_{z}$, and $O_{z}$. For illustrating the stimuli, we used a 21.5-inch LED screen with resolution of 1920×1080, and refresh rate of 60 Hz. The stimuli is shown over a white background while each participant was sitting at a distance of 70 cm from the screen.

Experimental Protocol: We used separate runs to test performance of the proposed DF-SSmVEP against the following two different paradigms: (i) Radial zoom, and; (ii) Rotation. Please refer to Reference [23] for videos of the test settings. Each video consists of five targets oscillating with different frequencies. For individual videos, target frequencies were 5, 6, 7, 8, and 9 Hz. For aggregated videos, target frequencies of the radial zoom pattern were 5, 6, 7, 8, and 9 Hz, and target frequencies of the rotation pattern were 5.5, 6.5, 7.5, 8.5, and 9.5 Hz. In each run, we included two consecutive sessions, in each of which the participants are asked to look directly at the target object identified with a pointer. During each session, we point to each of the underlying targets over four trials, where each of these four trials lasts for 3.5 s. A 2.5 s break is considered between each run of the consecutive trials. For evaluation purposes, ITR is used, which assesses the speed of a BCI systems as

(10) $$\begin{array}{c} \mathrm{ITR}=\frac{60}{T}\left[\log_{2}K+\sigma \log_{2}\sigma +\left(1-\sigma \right)\log_{2}\left(\frac{1-\sigma }{K-1}\right)\right], \end{array}$$

where σ denotes the recognition accuracy; *K* denotes the number of stimuli, and; *T* represents a value computed by adding the time of the resting state and the trial time. Evaluation via the One-way Analysis Of Variance (ANOVA) [24] with Tukey post hoc analysis is also used to confirm that responses to the proposed DF-SSmVEP-stimuli is statistically meaningful (*p* < 0.05). Some of the functions of [25] are used in the pipeline code. We would like to mention that one-way ANOVA (which can be considered to be the parametric equivalent of the Kruskal–Wallis test) is the common approach used in the literature to measure the significance of the VEP stimuli as such and for consistency is used in our study. Alternative non-parametric tests could be used for further analysis but heteroscedasticity of data (i.e., different groups have different standard deviations) should be carefully taken into consideration.

### 3.2. Results

**Figure 3:**
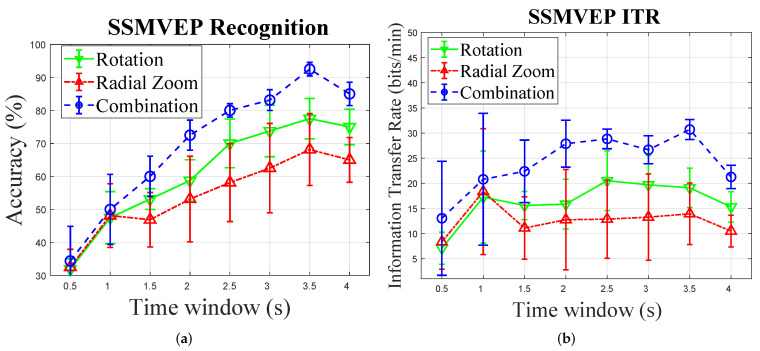
Mean and standard deviation across all subjects for each time window: (**a**) Accuracy comparisons. (**b**) ITR comparisons.

As shown in Figure 3, accuracy and ITR are measured for different time windows for each trial ranging from 0.5 to 4 s with an interval of 500 ms. The highest rate of transmission (ITR) belongs to aggregated motion (30.7 ± 1.97), which also achieved the best accuracy (92.5 ± 2.04). We used the ANOVA test following the existing literature [18,20] that used ANOVA test for significance comparison between accuracies and/or ITRs of different paradigms. It is worth noting that based on the Central Limit Theorem, we can safely assume that the samples have a normal distribution. The one-way ANOVA on accuracies of DF-SSmVEP paradigm reveals that there is no significant effect of frequencies (classes) on accuracies (F = 2.78, *p* = 0.065), so all target frequencies of DF-SSmVEP are feasible in BCI systems. The Tukey post hoc test on the accuracy and the ITR shows significant differences between the performance of DF-SSmVEP with BCCA and other paradigms with corresponding classifiers for which the highest accuracies are acquired, i.e., (Accuracy: $p_{DF-R}$ = 0.042, $p_{DF-RZ}$ = 0.002, $p_{R-RZ}$ = 0.218; ITR: $p_{DF-R}$ = 0.011, $p_{DF-RZ}$ = 0.001, $p_{R-RZ}$ = 0.264).

**Figure 4:**
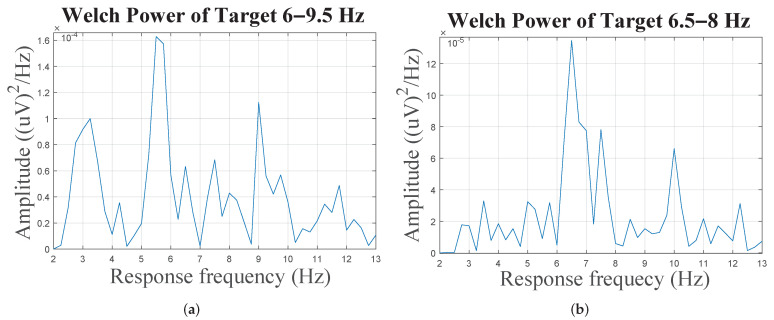
(**a**) The PSD plot based on aggregated SSmVEP with 6–9.5 Hz target frequencies. (**b**) Similar to (**a**) but with 8–6.5 Hz target frequencies.

Table 1 shows the overall recognition accuracies and ITRs. It can be observed that aggregating two SSmVEP paradigms using the proposed DF-SSmVEP results in compelling performance improvement. The results also show superiority of the BCCA as the best unsupervised target detector among its counterparts. Figure 4 illustrates Welch PSD of two targets of aggregated motion after spatial filtering for Subject 1. Two significant peaks of SSmVEP frequency around the two frequencies of each target is observable. Generally speaking, the Welch PSD is used to estimate random signals by dividing the data with a length of *N* into *M* segments of length *L*. The formula to compute the used window averaged period is given by $P\left(w\right)=1/M\sum_{i=1}^{M}[\frac{1}{LP_{0}}|\sum_{n=1}^{L}w\left(n\right)x_{i}\left(n\right)e^{-jwn}{|}^{2})]$, where $p_{0}$ refers to the power of window w(n), which is given by $P_{0}=1/L\sum_{n=1}^{L}{\left|w\left(n\right)\right|}^{2}$. According to [26,27], using welch PSD for detecting SSVEP and SSMVEP harmonics within EEG signal is more effective compared to Short-Time Fourier Transform (STFT) and filter banks. The Welch method reduces noise in the estimated power spectra in exchange for reducing the frequency resolution. Due to the presence of noise caused by EEG artifacts, the noise reduction from Welch’s method is indeed desired. Although the length of a trial is short, the target frequency and visual frequency band is limited to 20 Hz, which means a complete cycle of oscillation takes at most 50 ms that is much less than the trial length. Hence, the band-power of signal contains signals’ power, therefore, the superposition of sub powers will intensify the signal power and reduce noise power.

Mean and standard deviation based on four different Performance Indices (PI), i.e., precision, sensitivity, specificity, and accuracy across all runs for each class, are also shown in Table 2. In these calculations, the time window is set to 3.5 s. Each row of Table 2 corresponds to the dual frequency of the proposed DF-SSmVEP paradigm in each class. To investigate the robustness of the proposed methodology, we follow the common approach, which is to show paradigms repeatedly to different subjects (in contrast to using EEG signals of one trial several times) and average the performance across Runs and Subjects. As mentioned in the experimental protocol, in each run, we included two consecutive sessions, in each of which the participants are asked to look directly at the target object identified with a pointer. During each session, we point to each of the underlying targets over four trials. Hence, for each subject, the dataset includes 40 multidimensional signals associated with five classes. Each class represents target frequencies used in the corresponding motion paradigm where the cursor points to during data session. For example, if a subject stares at the rotation paradigm oscillating with 5 Hz and the cursor points to it, the EEG signal collected in this period of time will be labeled by 5 Hz. These labels are considered to be the ground truth for the dataset. BCCA and CCA also predict target frequencies used within a modulation for a given EEG signals. In this prediction task, the ratio of true positives (EEG signals predicted by correct target frequencies) over total records represents the accuracy. More specifically, in Table 2, specificity, sensitivity, accuracy, and precision of prediction for each class are measured as follows:

(11) $$\begin{array}{ccc} \mathrm{Sensitivity} & = & \frac{\mathrm{True}\;\mathrm{Positive}}{\mathrm{True}\;\mathrm{Positive}\;+\;\mathrm{False}\;\mathrm{Negative}} \end{array}$$

(12) $$\begin{array}{ccc} \mathrm{Specificity} & = & \frac{\mathrm{True}\;\mathrm{Negative}}{\mathrm{True}\;\mathrm{Negative}\;+\;\mathrm{False}\;\mathrm{Positive}} \end{array}$$

(13) $$\begin{array}{ccc} \mathrm{Precision} & = & \frac{\mathrm{True}\;\mathrm{Positive}}{\mathrm{True}\;\mathrm{Positive}\;+\;\mathrm{False}\;\mathrm{Positive}} \end{array}$$

(14) $$\begin{array}{ccc} \mathrm{Precision} & = & \frac{\mathrm{True}\;\mathrm{Positive}\;+\;\mathrm{True}\;\mathrm{Negative}}{\mathrm{Total}\;\mathrm{number}\;\mathrm{of}\;\mathrm{predictions}} \end{array}$$

where “True Positive” for a given class is the number of EEG signals labeled with name of that class correctly; “True Negative” for a given class is the number of EEG signals labeled with the name of other classes correctly; “False Positive” for a given class is the number of EEG signals labeled with the name of that class incorrectly, and; “False Negative” for a given class is the number of EEG signals labeled with the name of the other classes incorrectly.

## 4. Conclusions

To address lower accuracy and ITR of SSmVEP designs, this paper proposed an intuitively pleasing, novel, and innovative dual frequency aggregated modulation paradigm. Referred to as the DF-SSmVEP, the novel design is constructed by concurrently integrating “Radial Zoom” and “Rotation” motions in a single target without increasing the trial length. The paper also develops a specific unsupervised classification model, referred to as the BCCA, which uses availability of two motion frequencies per each target. While conventional Radial Zoom [19] and Rotation paradigms [19] have been used previously to design SSmVEPs, the proposed DFSSmVEP is developed by simultaneous combination (use) of these two conventional paradigms jointly. In other words, the DFSSmVEP is the first SSmVEP paradigm of its nature to concurrently use two different motions in one paradigm. Additionally, the frequency modulation and allocation of harmonics within accessible frequency bandwidth (visual bandwidth) is another novel characteristic of the DFSSmVEP. This is a unique way of associating different harmonics to different motion modes in one motion paradigm, which can be considered to be a step forward toward combining more SSmVEP patterns to develop new SSmVEP-based stimuli for evoking harmonic signals more strongly. The proposed DF-SSmVEP is evaluated via a real EEG dataset achieving average ITR of 30.07 ± 1.97 and average accuracy of 92.5 ± 2.04. As a final note, we would like to mention that it is well-known that EEG signals have a highly non-linear nature, the nature of non-linearity may even vary from subject to subject or even session to session. Although CCA has been widely used for comparing SSmVEP paradigms, its non-linear counterparts could be considered alternatively to relax the assumption of linear noise for removing artifacts to potentially improve the overall performance of the paradigm. In particular, advanced Deep Neural Networks (DNN) (e.g., similar in nature to the ones developed in our prior work [28]) can be designed to learn the type of non-linearity for each subject and remove it accordingly. However, such an approach, typically, is supervised needing a training phase making unsupervised CCA approach, although less accurate, more appealing. Development of semi-supervised or self-supervised DNN-based methods seem to be an interesting venue to pursue as a future direction. 

## Figures and Tables

**Table 1:** Mean accuracy (%) and mean ITR (bits/min) comparison between four methods of spatial filtering across the three motions. The best ITR among different time windows are reported for each filter. Maximum Contrast Fusion (MCF) [20] and T-F Image Fusion [17] are two types of spatial filtering.

	Paradigms	Radial Zoom		Rotation		DF-SSmVEP	
Filters							
		**ACC**	**ITR**	**ACC**	**ITR**	**ACC**	**ITR**
MCF + CCA		**68.12**	**18.35**	**77.5**	**20.52**	81.88	21.89
T-F Image Fusion + CCA		59.3	13.39	68.75	13.73	63.25	13.93
CCA Fusion		63.5	17.24	76.17	18.05	84.38	23.73
BCCA Fusion		-	-	-	-	**92.5**	**30.7**

**Table 2:** Comparison between mean and standard deviation values associated with performance indices (precision, sensitivity, specificity, and accuracy across) all run for three paradigms, i.e., Rotation (R), Radial Zoom (RZ), and the proposed DF-SSmVEP (denoted by DF).

	PI	Specificity			Sensitivity			Precision			Accuracy		
Classes													
		**R**	**RZ**	**DF**	**R**	**RZ**	**DF**	**R**	**RZ**	**DF**	**R**	**RZ**	**DF**
9 Hz or		0.912	0.934	**0.993**	**0.900**	0.600	0.875	0.735	0.710	**0.977**	0.910	0.867	**0.970**
(9 Hz, 7.5 Hz)		±0.052	±0.037	±**0.013**	±0.098	±0.226	±**0.083**	±0.137	±0.061	±**0.047**	±0.054	±0.020	±**0.01**
6 Hz or		0.940	0.893	**0.984**	0.725	0.875	**0.962**	0.757	0.702	**0.947**	0.897	0.890	**0.980**
(6 Hz, 9.5 Hz)		±**0.023**	±0.062	±0.027	±0.098	±**0**	±0.060	±**0.091**	±0.167	±0.870	±0.027	±0.050	±**0.023**
5 Hz or		**0.946**	0.875	0.940	0.625	0.937	**0.975**	0.737	0.663	**0.829**	0.882	0.8875	**0.952**
(5 Hz, 8.5 Hz)		±**0.025**	±0.044	±0.033	±0.220	±0.106	±**0.053**	±0.118	±**0.068**	±0.100	±0.054	±**0.017**	±0.029
7 Hz or		0.981	0.993	**0.993**	0.787	0.312	**1**	0.937	0.933	**0.977**	0.942	0.857	**0.995**
(7 Hz, 5.5 Hz)		±0.030	±0.013	±**0.013**	±0.177	±0.135	±**0**	±0.100	±0.140	±**0.047**	±0.026	±0.031	±**0.011**
8 Hz or		0.940	0.906	**0.987**	**0.850**	0.687	0.812	0.809	0.649	**0.939**	0.922	0.862	**0.952**
(8 Hz, 6.5 Hz)		±0.047	±0.062	±**0.016**	±**0.098**	±0.244	±0.135	±0.131	±0.221	±**0.079**	±**0.027**	±0.095	±0.036

## Data Availability

The anonymized dataset created for this research work is available through the following page: https://github.com/raykakarimi/DFSSMVEP (accessed on 19 January 2022).
